# Supplementary figures and images for: Validation and Determination of 25(OH) Vitamin D and 3-Epi25(OH)D3 in Breastmilk and Maternal- and Infant Plasma during Breastfeeding
Source: Nutrients. 2020 Jul 29;12(8):2271. doi: 10.3390/nu12082271 (PMC7469027; doi:10.3390/nu12082271)

## Supplementary material

**Figure A.** Representative chromatograms of 25(OH)D3 and 3-Epi-25(OH)D3 8 (a.) and 25(OH)D2 (b.).

a.

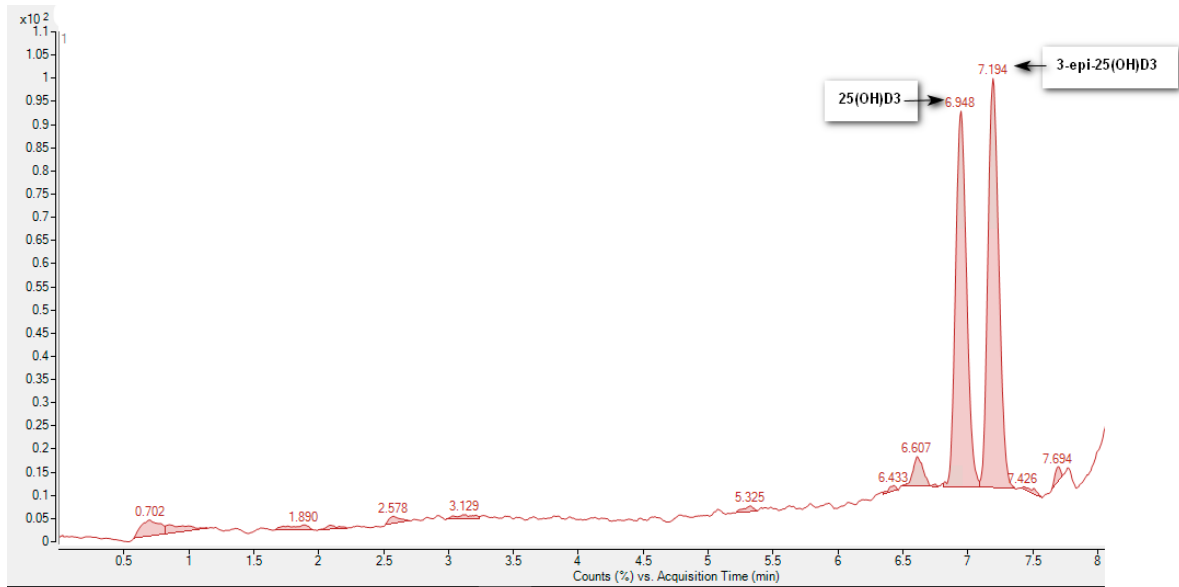

b.

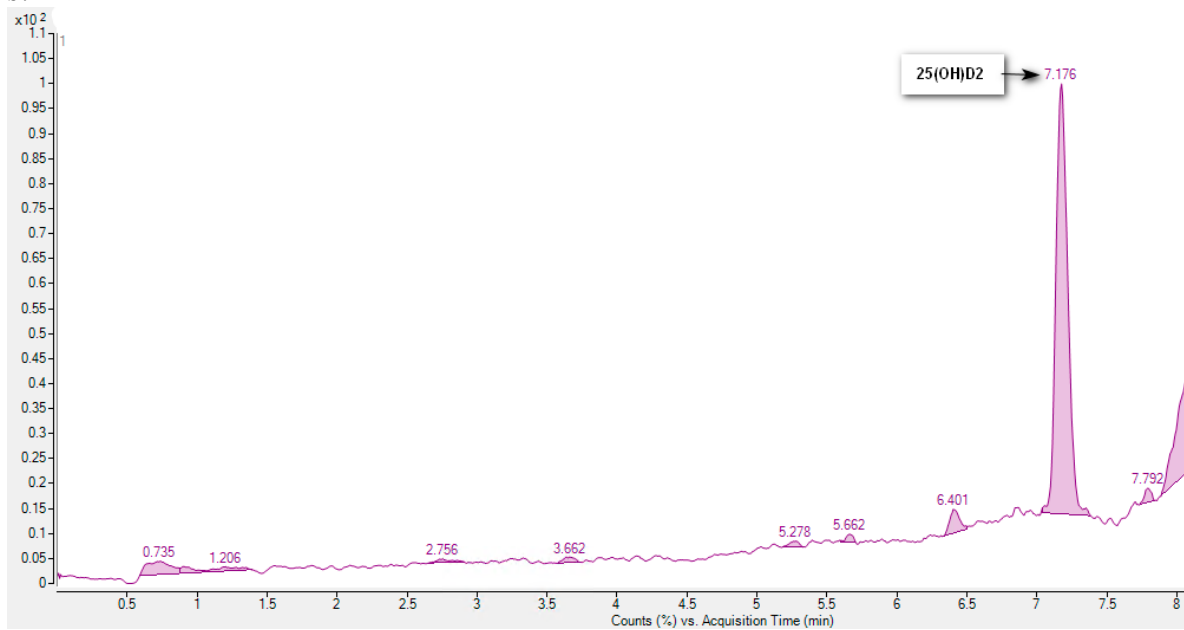

Supplement: Supplementary file 1 [file nutrients-12-02271-s001.pdf]
